# Isolation and Identification of Four Strains of Bacteria with Potential to Biodegrade Polyethylene and Polypropylene from Mangrove

**DOI:** 10.3390/microorganisms12102005

**Published:** 2024-10-02

**Authors:** Xilin Fang, Zeming Cai, Xiaocui Wang, Ziyu Liu, Yongkang Lin, Minqian Li, Han Gong, Muting Yan

**Affiliations:** College of Marine Sciences, South China Agricultural University, Guangzhou 510642, China; fangxl1133@163.com (X.F.); 13172520909@163.com (Z.C.); cc45683968exo@163.com (X.W.); 18590187294@163.com (Z.L.); lyk20020404@163.com (Y.L.); koitoyuu045@foxmail.com (M.L.)

**Keywords:** polyethylene (PE), polypropylene (PP), microplastics, biodegradation, mangrove

## Abstract

With the rapid growth of global plastic production, the degradation of microplastics (MPs) has received widespread attention, and the search for efficient biodegradation pathways has become a hot topic. The aim of this study was to screen mangrove sediment and surface water for bacteria capable of degrading polyethylene (PE) and polypropylene (PP) MPs. In this study, two strains of PE-degrading bacteria and two strains of PP-degrading candidate bacteria were obtained from mangrove, named *Pseudomonas* sp. strain GIA7, *Bacillus cereus* strain GIA17, *Acinetobacter* sp. strain GIB8, and *Bacillus cereus* strain GIB10. The results showed that the degradation rate of the bacteria increased gradually with the increase in degradation time for 60 days. Most of the MP-degrading bacteria had higher degradation rates in the presence of weak acid. The appropriate addition of Mg^2+^ and K^+^ was favorable to improve the degradation rate of MPs. Interestingly, high salt concentration inhibited the biodegradation of MPs. Results of scanning electron microscopy (SEM), atomic force microscopy (AFM), and Fourier-transform infrared spectroscopy (FTIR) indicated the degradation and surface changes of PP and PE MPs caused by candidate bacteria, which may depend on the biodegradation-related enzymes laccase and lipase. Our results indicated that these four bacterial strains may contribute to the biodegradation of MPs in the mangrove environment.

## 1. Introduction

Plastic products are widely utilized due to their excellent durability, resistance to corrosion, mechanical integrity and strength, flexibility, and insulating qualities [1]. With the rapid increase in worldwide plastic production, a substantial number of plastic products are not properly disposed of and end up in landfills or the natural environment [2]. As of 2016, it was anticipated that 150 MMT of plastic were present in the maritime ecosystem [3]. Of the total global use of plastics, about 80% is petrochemical plastics such as polyethylene (PE), polypropylene (PP), and polyvinyl chloride (PVC) [4]. However, typical petroleum-based plastics degrade over time due to abiotic causes (UV radiation, temperature, physical stress), resulting in microplastics (MPs) that are not completely broken down and ingested by microorganisms (organisms) during biodegradation [5]. Plastic can linger in the ocean in its original form for hundreds of years, or even longer in the form of tiny particles (i.e., microplastics). Therefore, determining how to reduce the level of MPs has become a hot issue.

Two methods that are being used for MP breakdown are biological degradation and advanced oxidation processes (AOPs). AOPs, a chemical elimination technology, effectively degrades persistent organic contaminants in water, which can lead to chain breaks, formation of useful products, or even complete mineralization of MPs [6]. Apart from AOPs, bioremediation was also thought to be the best approach to eliminate MP pollution. The use of certain microorganisms improves the biodegradation efficiency of MPs [7]. This offers a sustainable and effective way to accelerate MP biodegradation with little negative impact on natural ecosystems [8]. In general, biodegradation consists of two critical steps. First, the polymer is broken down into its constituent monomers, which are subsequently mineralized. The molecular weight of plastic is reduced, making it easier to pass through cell membranes and intracellular enzymes. Next, the tricarboxylic acid cycle and β-oxidation break down hydrocarbons into carboxylic acids, and eventually CO_2_, H_2_O, or CH_4_ are produced [8,9]. Recently, some research has looked into the employment of microbes to degrade MPs. PE MPs could be used by *Zalerion maritimum* (Ascomycete) and the quality and size of the pellets (minimum nutrient requirements) were reduced [10]. Bacillus strain 27 and Rhod coccus strain 36 from mangrove sediments could degrade PP MPs with altered mass and morphology [11]. LDPE decreased in size under *Bacillus simplex* and *Bacillus* sp. treatment and released different volatiles during the treatment [12].

Mangrove forests, which cover 0.5% of the coastal areas of the world, are primarily found in the intertidal zones of subtropical and tropical coasts. Additionally, they are well-known as key pollutant buffers between land and sea [13,14]. Mangroves intercept MPs from different sources via complex methods (e.g., riverine, atmospheric, and marine MPs) [15,16,17]. Due to the fact that most mangrove forests grow where they are not affected by waves and the plants are a barrier to wave action and water currents, once the MP enters the mangrove forests, the portion that is denser than seawater (e.g., PS, PVC, and PET) will be deposited directly into the sediments, while the low-density microplastics float on the surface of the water [18]. According to statistics, microplastics make up approximately 3.3% of the weight of silt [19], with an average of 851 ± 177 microplastic particles per kilogram of sediment observed in mangrove sediments along the coast of South China [20]. Furthermore, mangrove sediments in the northern Persian Gulf were found to contain an average number of 34.5 ± 19.5 isolated microplastics [21], while mangrove sediments in Singapore were found to contain 23.6 ± 36.8 particles per kilogram of sediment due to microplastic contamination [22]. In the surface water of the mangrove forests at the different sites, there were 177 to 76 MPs/L in an urban (Durban Bay) and peri-urban (Mngazana Estuary) mangrove forest in South Africa [23]. The number of microplastics in surface water was 81,056 ± 8085 microplastics/km^2^ in the pre-monsoon and 73,722 ± 9096 microplastics/km^2^ in the post-monsoon in the mangrove Sundarbans in the northern Bay of Bengal off the coast of Bangladesh [24]. In the tropical mangroves of Kota, South-western India, mean concentrations (±standard deviation) of 1.42 ± 0.92 MPs/L (post-monsoon), 0.62 ± 0.38 MPs/L (pre-monsoon), and 0.19 ± 0.08 MPs/L (monsoon) were found [25]. There are numerous microplastics found in mangrove seawater and sediments. Mangrove clogging of MPs may lead to changes in the physicochemical composition of mangrove seawater and sediments (e.g., water quality, porosity, aggregates, bulk density, and functional groups) [26], which can reduce the diversity and abundance of bacterial communities [27], as well as being ecotoxic to mangrove organisms [28,29].

The mangrove microbiota consists of terrestrial, marine, and freshwater microorganisms, and they play essential roles in the mangrove ecosystem functioning together with ecosystem maintenance [30]. Numerous bacterial species were found to be flourishing on the surface of PE in water and sediment, according to studies conducted in mangrove ecosystems [31]. Towards this, we sampled sediments and water from the mangrove forests of Gull Island, Guangzhou City, Guangdong Province, China to screen for PE and PP microplastic-degrading microorganisms and to characterize potentially efficient PE and PP microplastic-degrading bacteria. The aims of this study are to: (1) screen and characterization of two different types of microplastic-degrading bacteria for PP and PE MPs; (2) investigate the microbial degradation characteristics of PP and PE MPs; and (3) briefly clarify the degradation mechanisms of potentially efficient PP and PE microplastic-degrading bacteria.

## 2. Materials and Methods

### 2.1. Samples and Microplastics

This study collected soil samples (0–2 cm) from Gull Island in Guangzhou, Guangdong, China, as well as water samples from the same location (22°54′20″ N, 113°32′26″ E). Two different kinds of microplastics—PE and PP MPs—were acquired for this investigation from Wanda (Dongguan) Plastic Material Co. (Dongguan, China). It was oval-shaped and measured about 3 mm and 300 mesh (about 50 microns). Prior to conducting the studies, two types of microplastics, PE and PP MPs, were agitated for four hours while submerged in a 1% sodium dodecyl sulfate solution (SDS). Next, the SDS that had adhered to the surface of the microplastics was cleaned with sterile water. Subsequently, 75% alcohol was added to PE and PP microplastics, which were then mixed for over 4 h. The alcohol was then emptied, coated with tin foil paper, and dried at 60 °C. After it was dried, it was set on the spotless workstation, and the UV lamp was turned on for 30 min.

### 2.2. Bacterial Culture

Two enrichments were carried out to improve the survival of microbes that rely solely on microplastics for carbon as well as to screen for bacteria that can survive just on plastics. The soil or water, 10% (*w*/*v*), and sterile PE and PP microplastics, 1% (*w*/*v*), were cultivated in 200 mL of microcarbon medium (pH 7.0). The mixture was subsequently left to incubate at 28 °C with shaking at 180 rpm/min for 20 days. The second enrichment was carried out in 200 mL of inorganic salt medium (pH 7.0). The PE and PP microplastics after the first enrichment culture in the microcarbon source medium were separated by a sieve, added into the sterilized inorganic salt medium, and incubated in a shaker at 180 rpm and 28 °C for 30 days. The microcarbon composition of the microcarbon medium is shown in Table S1. The inorganic salt composition of the inorganic salt medium is shown in Table S2.

### 2.3. Bacterial Isolation and Identification

We aspirated 100 μL of bacterial solution from the inorganic salt medium after 30 days of incubation in Section 2.2, performed a gradient dilution, and applied 10^−1^ to 10^−6^ concentrations sequentially to LB agar medium. The plates were incubated in a constant temperature incubator at 28 °C for 12–16 h until single colonies visible to the naked eye grew on the plates. The 10^−3^ concentration of coated plates was used for the isolation and purification of degradative bacteria in this experiment based on the quantity and size of the colonies. The isolated strains were collected and stored at −80 °C. The approach for sequencing 16S rDNA followed prior research procedures [32,33]. The 16S rDNA sequence was cloned by PCR using primers 16sbac27F-AGA GTT TGA TCM TGG CTC AG and 16sbac1492R-TAC GGY TAC CTT GTT ACG ACT T and processed in a thermal cycler. The sequences were identified in the NCBI database. 

### 2.4. Related Enzymes and Enzyme Activity Assays 

Laccase, urease, lipase, H_2_S, and H_2_O_2_ production tests, the color changes, and colony shape features that the medium produced were noted and examined. The specific experimental information is shown in Table S3. 

Laccase and lipase are frequently claimed to have a role in plastic degradation [34], hence we investigated their activities. Laccase and lipase activities were determined using the criteria provided in the Laccase Activity Assay Kit (Solarbio, Beijing, China) and Lipase (LPS) Activity Assay Kit (Solarbio, China), respectively.

### 2.5. Weight Loss of PE and PP MPs

The potentially effective PE and PP microplastic-degrading bacteria were screened based on the findings of physiological and biochemical tests as well as the identification of species of microplastic-degrading bacteria. Ten percent of the volume of the bacterial solution and the appropriate amount of PE and PP microplastics were added to the inorganic salt medium, and the incubation was conducted for 60 days in a shaker at 180 rpm and 28 °C. The same conditions as a blank control were used for the incubation of the PE and PP microplastic-inorganic salt medium without the inclusion of a bacterial solution. After 60 days, the PE and PP microplastics in the inorganic salt medium were taken out, put into 1% SDS solution and stirred for 4 h, washed with sterile water, and then soaked in 75% alcohol and stirred for more than 4 h. After drying at 60 °C, the microplastics were weighed and the degradation rate of microplastics was calculated. The weight loss of microplastics was analyzed gravimetrically using the degradation rate as an indicator. Untreated PE and PP microplastics were determined as *W*0. The weight of PE and PP after 60 d of incubation was determined as *W*1. The microplastic degradation rate was calculated using the following formula:

$$weight\;loss\;\left(\%\right)=\frac{\left(W0-W1\right)}{W0}\times 100\%.$$

### 2.6. Degradation Rates of Potentially Efficient Microplastic-Degrading Bacteria under Different Cultural Conditions

The degradation rate of potentially efficient microplastic-degrading bacteria was investigated by varying the period, pH environment, inorganic salt ions, and salt concentration [35].

To the autoclaved inorganic salt medium, 10% volume of bacterial solution and weighed PE or PP microplastics (3 mm, 1%, *w*/*v*) were added. Then, OD_600_ was tested and incubated for 30, 40, and 60 days at a shaker temperature of 28 °C and 180 rpm. On the 30th, 40th, and 60th days, the medium of the experimental group at that time was taken out to test the OD_600_, and the PE or PP microplastics of the group were taken out. Following procedure 2.5 of washing and drying, the PE and PP microplastics were weighed and recorded, and the formula was used to determine the rate of microplastic disintegration.

The same techniques were used to ascertain the rate of microplastic disintegration following a 60-day incubation period in various pH conditions. This was accomplished by adding sodium hydroxide and hydrochloric acid to the designed inorganic salt medium dropwise until the pH levels were 5, 6, and 8, accordingly. Following 60 days of incubation under various inorganic salt ions, the degradation rate of microplastic was evaluated by incorporating 0.3% of magnesium sulfate (MgSO_4_), and dipotassium hydrogen phosphate (K_2_HPO_4_) into the inorganic salt medium. Adjusting the proportioning concentration of NaCl to 1 g/L, 2 g/L, and 3 g/L while keeping the proportioning of other components constant, the degradation rate of microplastics was determined and calculated after 60 days of incubation. 

### 2.7. Characterization of MPs before and after Biodegradation

#### 2.7.1. Scanning Electron Microscopy (SEM) Analysis

After 60 days of incubation, PP and PE MPs (3 mm and 700 μm) were removed from the media to measure surface erosion and degradation. One percent sodium dodecyl sulfate (SDS) and distilled water were used to wash the microplastics (3 mm). Prior to plating, the samples were dried at 60 °C in an oven, and pictures were captured using an EVO MA 15 scanning electron microscope (Zeiss, Jena, Germany). In contrast, the 700 μm PP and PE MPs were dehydrated in a gradient from 30% to 100% ethanol after being fixed with 2.5% glutaraldehyde and 0.5% osmium acid. Saturated and dehydrated microplastics were produced using a critical point dryer (CPD 300, Leica, Wetzlar, Germany) and then coated with gold spraying to observe the biofilm on the microplastic surface under a field emission scanning electron microscope (Gemini 500, ZEISS, Germany).

#### 2.7.2. Fourier-Transform Infrared Spectroscopy (FTIR) Analysis

After 60 days of bacterial treatment, both treated and untreated PP and PE MPs were rinsed with 1% SDS and dried at 60 °C. All samples were analyzed by FTIR using a Fourier-transform infrared spectrometer (Nicolet IS10, Thermo Fisher Scientific, Waltham, MA, USA) at 4000–400 cm^−1^ and scanned 32 times. Baseline mode was used to depict the spectra.

#### 2.7.3. Atomic Force Microscopy (AFM) Analysis

For AFM investigations, 300 mesh (~50 μm) PE/PP microplastics were used due to sample size constraints of the instrument. After 60 days of incubation with the bacterial isolates, PP and PE MPs were taken out of the medium, filtered through a 300-mesh screen, and subjected to the previously mentioned SEM processing. The increase in roughness of the microplastic surface was observed by AFM (SPM9700, SHIMADZU, Kyoto, Japan) with a scanning size of 5 × 5 μm.

### 2.8. Data Analysis

The degradation rate and OD_600_ change of microplastics were given as mean ± S.E. Data processing for this study was done using Excel 2021, while graphs and other visualizations were created using Origin 2021. The sequences of target strains were analyzed using MEGA 7 to generate a Neighbor-Joining tree (Bootstrap is 1000).

## 3. Results and Discussion

### 3.1. Isolation and Identification of Microplastic-Degrading Bacteria

**Figure 1:**
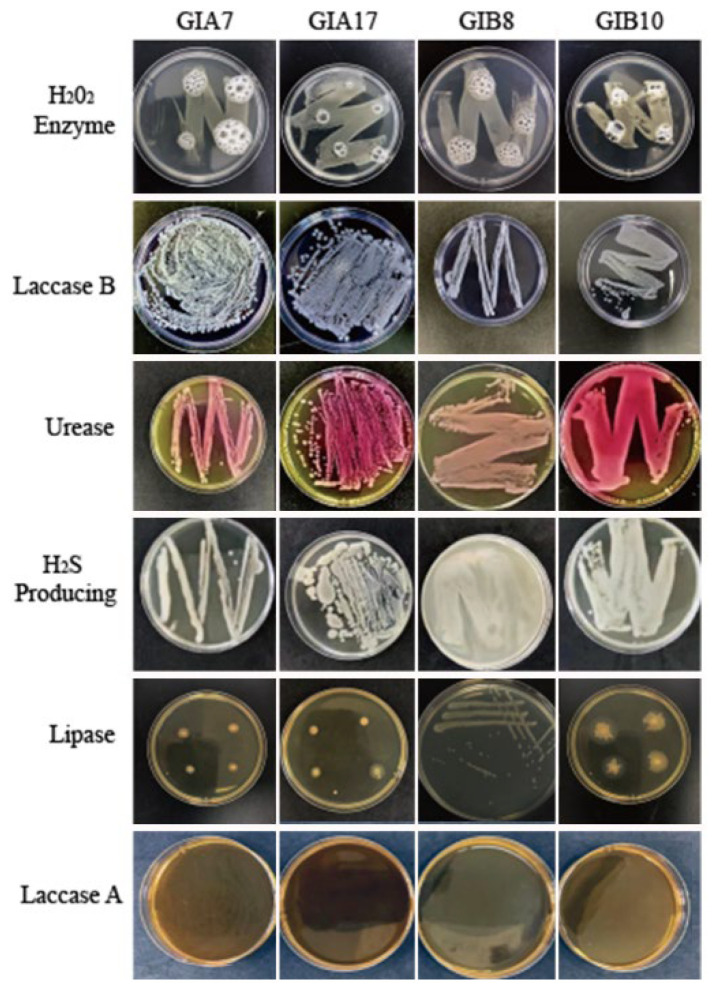
Results of physiological and biochemical experiments on potentially efficient microplastic-degrading bacteria.

After enrichment by the microcarbon medium, 30 strains each of PP and PE MP-degrading bacteria were selected for physiological and biochemical tests. The second stage of microbial degradation of plastics is the secretion of extracellular enzymes from microbial biofilms that are involved in the oxidation and depolymerization of microplastics [36]. Enzymes such as laccase, lipase, peroxidase, and urease are reported to be involved in the degradation pathway of microplastics [37,38]. Enzyme activity tests are physiological and biochemical tests to detect whether potential degrading bacteria are able to produce these enzymes, thus further determining their degradability [39]. The results showed that no color change was observed in laccase A, suggesting that laccase A was not generated by candidate bacteria. No color change was observed in the H_2_S test, suggesting that bacteria were unable to decompose sulfur-containing organic matter. On the contrary, H_2_O_2_, laccase B, urease, and lipase culture have the corresponding changes: dropping H_2_O_2_ rapid production of bubbles, laccase B blue color fading, urease producing a large number of red, as well as lipase colonies around the smaller transparent circle (Figure 1). Based on the medium indicator reaction (Table S4), 12 strains of PP MP-degrading bacteria and 18 strains of PE MP-degrading bacteria with medium indicator changes were selected. 

**Figure 2:**
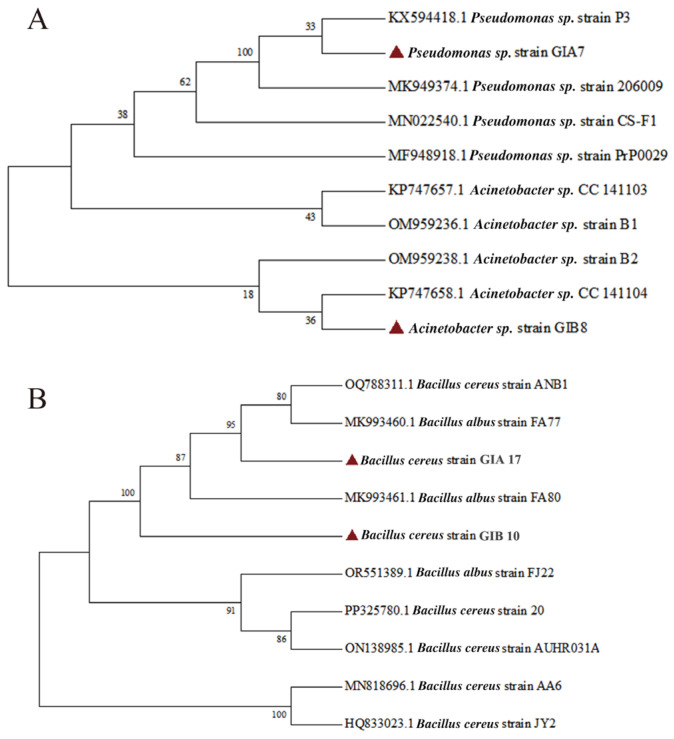
Phylogenetic tree indicating the relationship between the 16S rRNA gene sequences of (**A**) *Pseudomonas* sp. strain GIA7 and Acinetobacter sp. strain GIB8, (**B**) Bacillus cereus strain GIA17 and Bacillus cereus strain GIB10. The red triangle signs are the four strains of potentially efficient degrading bacteria.

These 12 strains of PP MP-degrading bacteria and 18 strains of PE MP-degrading bacteria were sequenced using 16S rDNA amplification and analyzed in the NCBI database for homology comparison to determine the species of the strains. The microplastic-degrading bacteria isolated from seawater and soil samples from Guangzhou Gull Island were categorized as follows: *Pseudomonas* spp., *Bacillus* spp., *Pseudomonas mosselii*, *Pseudochrobactrum saccharolyticum*, *Brevundimonas faecalis*, and *Brevundimonas ole*, among others. Bacteria with multiple key degradative enzymes and non-duplicated species identification were selected for 60 days of inorganic salt medium culture with PE/PP microplastics as the sole carbon source. It has not been possible to biodegrade PE more than 50% or fully into biomass, CO_2_, water, and minerals up to this point utilizing biological processes or the ISO 17556, and other standards [40,41,42,43] for biodegradable plastic materials [44,45,46]. Therefore, according to the weight loss rate, GIB8, GIB10, GIA7, and GIA17 had relatively high weight loss rates. Four strains of potentially efficient microplastic-degrading bacteria showed different degrees of degradation (Figure S1), among which strain GIB8 showed the best degradation effect with a degradation rate of 0.32%, followed by GIB10, GIA7, and GIA17 with 0.28%, 0.15%, and 0.13%, respectively. The sequences of four strains were identified in the NCBI database, GIA17 and GIB10 belong to *Bacillus cereus*, GIA7 belongs to *Pseudomonas* sp., and GIB8 belongs to *Acinetobacter* sp. (Table 1). Thus, we named GIA7 as *Pseudomonas* sp. strain GIA7 (GIA7), GIA17 as *Bacillus cereus* strain GIA17 (GIA17), GIB8 as *Acinetobacter* sp. strain GIB8 (GIB8), and GIB10 as *Bacillus cereus* strain GIB10 (GIB10). According to the molecular gene analysis, strain GIA7 was similar (100%) to *Pseudomonas* sp. strain P3, strain GIB8 was similar (100%) to uncultured *Acinetobacter* sp., strain GIA17 was similar (99.33%) to *Bacillus cereus* strain B2023, and strain GIB10 was similar (99.18%) to *Bacillus cereus* strain ANB1. A phylogenetic tree of GIA7, GIA17, GIB8, GIB10, and related species is shown in Figure 2. Thus, two strains of PE-degrading bacteria, GIA7 and GIA17, and two strains of PP-degrading candidate bacteria, GIB8 and GIB10, were continued with subsequent experiments.

### 3.2. Weight Loss Rate of MPs under Different Conditions

**Figure 3:**
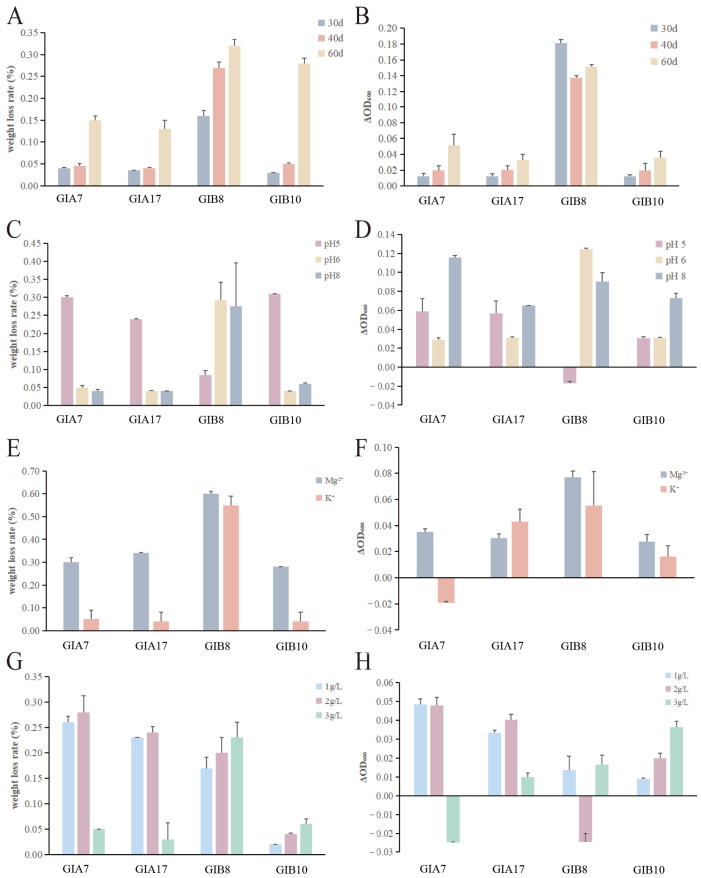
Weight loss rate of MPs after degradation by potentially efficient microplastic-degrading bacteria (**A**) at different times, (**C**) in different pH environments, (**E**) under different inorganic salt ions, (**G**) at different salt concentrations. Plot of ΔOD_600_ of potentially efficient microplastic-degrading bacteria (**B**) at different times, (**D**) in different pH environments, (**F**) under different inorganic salt ions, (**H**) at different salt concentrations.

The weight loss rate of PE/PP microplastics by the four strains of potentially efficient PE/PP microplastic-degrading bacteria increased with the increase in the number of days (Figure 3A). Overall, the degradation of microplastics by PP MP-degrading bacteria was better than that of PE MP-degrading bacteria, with the best degradation by GIB8, followed by GIB10 and GIA7, and the poorer degradation by GIA17. The higher ΔOD_600_ increment of strains with PP MPs as the sole carbon source than those with PE MPs as the sole carbon source (Figure 3B) could also indicate that PE MPs are more difficult to utilize by microorganisms than PP MPs.

Our results (Figure 3A) are comparable to those of previous studies that obtained weight reductions in PE (13.3%) and PP (32.6%) after three months of degradation, respectively [11,47]. According to the present study, the weight loss after 60 days of incubation amounted to 0.23% and 0.16% for GIA7 and GIA17, respectively. These results were much lower than the results of PE degraded for 56 days (7.50%). One possible explanation could be that the strains used in their study were more suitable for degrading PE, with the biofilm differentiation rate of the red mold strain C208 being significantly higher than that of *Pseudomonas aeruginosa* [48]. 

The pH can be altered to change the rate of the hydrolysis reaction by changing the acidic or alkaline conditions. Degradation products of various polymers alter the pH conditions and subsequently change the rate of the degradation process and microbial growth [49]. Therefore, we explored the weight loss rate of PE/PP MPs by the four potentially efficient PE/PP microplastic-degrading bacteria under different pH conditions, and they were significantly different (Figure 3C). All degrading bacterial strains favored acidic environments for growth. Consistent with previous findings, the rate of hydrolysis of PLA capsules was optimal at pH 5 [50]. Among all strains, GIA7, GIA17, and GIB10 were best degraded at pH 5. However, from the ΔOD_600_ values (Figure 3D), the growth values at pH 8 were higher than those at pH 5 and pH 6. In keeping with previous findings, the optimal condition for the growth of *Bacillus cereus* was at pH 7.4 [51], and *Pseudomonas mangrovi* sp. nov. isolated from the inter-root soil of a mangrove forest (*Kandeliaobovata*) in Fujian Province, China grew optimally at pH 7.5 [52]. This suggests that GIA7, GIA17, and GIB10 may be better adapted to grow in neutral environments, and better able to perform degradation in acidic environments. GIB8 had the highest weight loss rate at pH 6, followed by pH 8. Although the weight loss rates were close to each other at pH 6 and pH 8, the growth of the degrading bacteria at pH 6 was significantly better than that at pH 8 in terms of the ΔOD_600_ value. It indicated that the environment at pH 6 was more favorable for the growth of the degrading bacteria. In general agreement with previous findings, diesel-degrading bacterial strains isolated from a petroleum waste dump site showed optimal degradation efficiency at pH 7 (30 °C) [53], and optimal growth conditions for *Acinetobacter tibetensis* sp. nov. isolated from soil under a greenhouse in Tibet, China with iprodione-degrading ability also was pH 7 (25 °C) [54]. Due to the lack of studies at pH 7 conditions in this study, it is uncertain whether the strain exhibited optimal degradation efficiency at pH 7. It is hypothesized from the results that PP8 may show an increasing trend of degradation efficiency at pH 6–7 and a decreasing trend of degradation efficiency at pH 7–8. The above results indicate that GIA7, GIA17, GIB8, and GIB10 were adapted to degrade in an acidic environment.

Both Mg^2+^ and K^+^ are common ions in the water column and inorganic salt ions required for the growth of the strains. After adding additional inorganic salt ions of 0.3% Mg^2+^ or K^+^ (Figure 3E), most of the degrading bacteria had higher weight loss than those without inorganic salt ions. Among them, all of them had weight loss with Mg^2+^ added, except PE7. And the rate of weight loss was higher with Mg^2+^ added than with K^+^ added. The ΔOD_600_ before and after degradation with different inorganic salt ions (Figure 3F) also confirmed this result. The bacteria grew better in the medium with Mg^2+^ than K^+^. It indicated that the appropriate addition of Mg^2+^ and K^+^ can help improve the degradation effect of degrading strains, and it is possible that the bacteria need Mg^2+^ more during the degradation process. Consistent with the findings of prior investigations, the order of requirement for check cations in *Acinetobacter junii* was Mg^2+^ > K^+^ [55].

Salinity significantly alters the distribution of microplastic-degrading bacteria, and bacteria on the surface of MPs in rivers may lead to higher microplastic degradation than offshore [56]. With the increase of salt concentration, the weight loss rate of GIA7, GIA17 increased and then decreased (Figure 3G), and the weight loss rates were close to each other at the concentrations of 1 g/L and 2 g/L, and the weight loss rate decreased substantially at 3 g/L. However, the weight loss rate of GIB8 and GIB10 increased with increasing concentration, suggesting that GIB8 and GIB10 are more adapted to high salt environments in degrading PE and PP MPs. Their ΔOD_600_ values share the same trend (Figure 3H). Less consistent with previous findings, salinity was negatively correlated with MPs-attached bacteria in water, showing a clear downward trend from riverine to marine environments [57]. One possible explanation is that the mangrove forest from which the samples were collected is a land–sea interface, and it is possible that some of the bacteria are salt-tolerant while others are not.

### 3.3. Determination of Enzyme Activity of Potentially Efficient Microplastic-Degrading Bacteria

**Figure 4:**
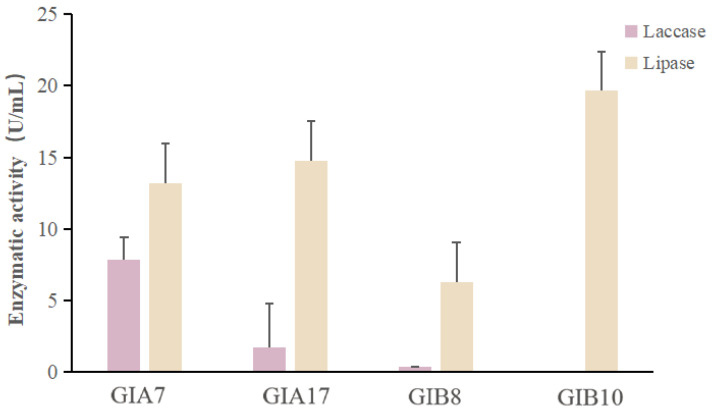
Laccase and lipase activities of the strains.

Microbes (mainly bacteria and fungi) frequently create extracellular enzymes that assist in degrading various types of bio- and fossil-based polymers [58]. The hydrolysis of the polymers can be facilitated by the released enzymes adhering to the surface of the microplastic. The enzymatic breakdown of the polymer produced tiny organic molecules that microbes can use as carbon sources [59]. Gaur et al. [60] observed that a variety of enzymes were released during the microbial degradation of microplastics, including manganese peroxidase, lipase, amidase, esterase, and laccase. Among them, lipase and laccase are often reported to play a role in plastic degradation [34], so we measured the activities of laccase and lipase to determine the degradation effect of the bacteria. Laccase is a copper-containing polyphenol oxidase belonging to the copper blue oxidase family, widely distributed in fungi and higher plants, with a strong redox ability in pulp bio-bleaching, environmental pollutants degradation, and lignocellulose degradation, as well as bio-detection, and has a very wide range of applications [61,62,63]. This experiment uses a laccase reagent kit, reagent laccase blank tube under normal circumstances, change in the OD value is less than 0.05, the experiment measured OD value is less than or equal to 0.048, and meets the requirements. Enzyme activity definition: per milliliter of liquid per minute oxidation of 1 nmol of substrate ABTS enzyme volume required for a unit of enzyme activity. After the test, laccase activity (nmol/min/mL) could be calculated by 37.04 × ΔA. Lipase is a special kind of ester-bonded hydrolyzing enzyme that catalyzes the hydrolysis of natural fats and oils and has a wide range of applications in many industrial fields such as food, medicine, detergent, and leather [64,65]. In this experiment, a lipase (LPS) activity kit was used with the standard curve equation: y = 0.0124x − 0.003; x is the molar mass of the standard (nmol) and y is ΔA. The lipase equation was calculated after the test y = −0.0016x + 0.2892, R2 = 0.9632. From the graphs of the activity of laccase and lipase (Figure 4), it was found that GIA7 had higher enzyme activity, while GIB10 was 0, indicating that the strain may not produce laccase. The enzyme activity of lipase was better than that of laccase, with all four strains higher than 5, and the highest GIB10 was 21. According to earlier research findings, the enzyme activity of lipase was much higher than that of laccase after 30 days of incubation [66].

### 3.4. Scanning Electron Microscopy Results before and after Microplastic Degradation

**Figure 5:**
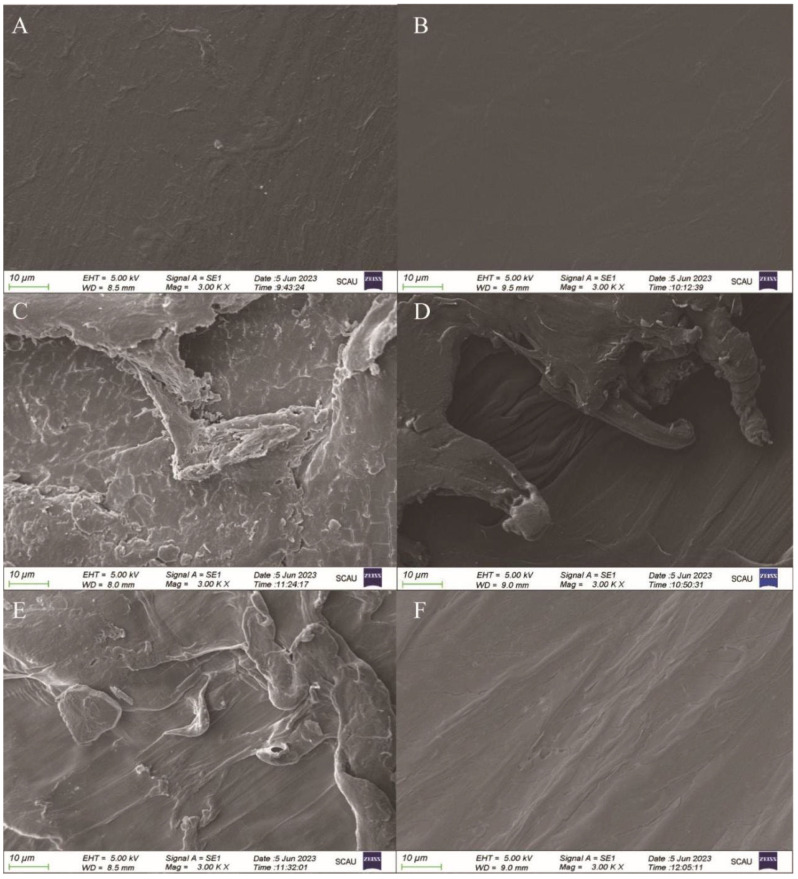
Surface microscopic characterization of PE and PP microplastics before and after degradation by scanning electron microscopy. Untreated PE microplastics (**A**) and untreated PP microplastics (**B**). The surface change of PE MPs after 60 days of degradation by GIA7 (**C**). The surface change of PE MPs after 60 days of degradation by GIA17 (**E**). The surface change of PP MPs after 60 days of degradation by GIB8 (**D**). The surface change of PP MPs after 60 days of degradation by GIB10 (**F**).

The microplastics after 60 days of degradation were washed, and after removing the surface impurities, the microscopic characteristics of MPs before and after degradation of different surfaces were observed by scanning electron microscopy to see whether the surfaces appeared to be characterized by pits, folds, and so on. The structure of PE MPs (Figure 5A) and PP MPs (Figure 5B) without microbial treatment did not show much difference. The surface was relatively smooth and basically free of wrinkles, and there were no visible pores or micro-damages under the magnification of the microscope. The microplastic surfaces have been subjected to the effects of enriched culture colonies and exhibit rough cracks and scratches. The surfaces of these microplastics were covered with flakes, pits, and grooves, which are good substrates for biofilm formation. Pits and grooves on the surface of PE MPs were relatively more obvious than those of PP MPs. After the degradation of GIA7 (Figure 5C) and GIA17 (Figure 5E), a large number of cracks appeared on the PE MPs surfaces, and the degree of breakage was larger. The appearance of pits and wrinkles on the PP MPs surfaces was more obvious, and after degradation of GIB8, the surface showed some degree of wrinkles, grooves, and other phenomena (Figure 5D), while slight folds were visible on the surface after degradation of GIB10 (Figure 5F). SEM images showed that colonization and the formation of biofilms were more pronounced on the surface of the mangrove bacteria-treated PP and PE MPs compared to the non-marine bacteria-treated PP and PE MPs. The surface deterioration, friability, broken layers, and obvious cracks and scratches of the mangrove bacteria-treated PP and PE MPs were evident in the SEM photographs. Notably, these erosion pits were absent from the original surfaces of MPs. Specifically, numerous deep holes and heavy cracks were observed on the PE MP surface, indicating signs of polymer biodegradation.

**Figure 6:**
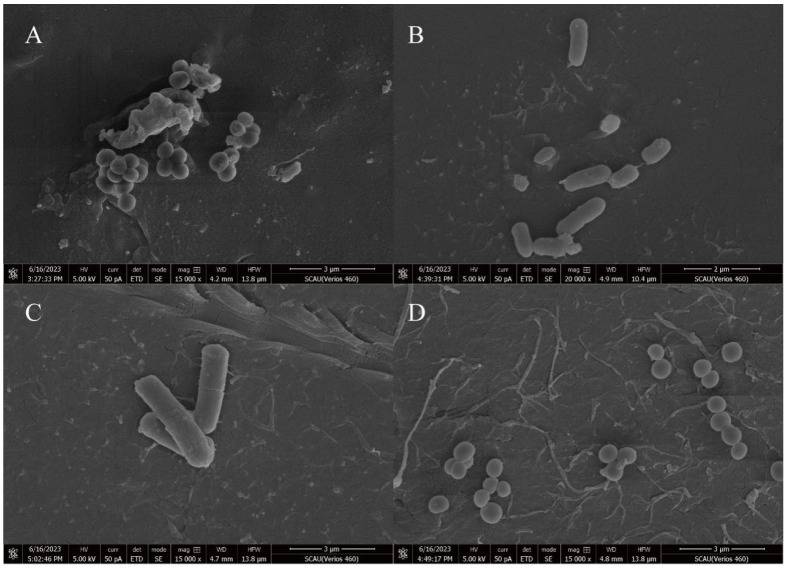
Bacterial attachment of PE and PP MPs observed using SEM after 60 days of incubation. PE MPs after degradation by GIA7 (**A**) and GIA17 (**B**). PP MPs after degradation by GIB8 (**C**) and GIB10 (**D**).

Microplastics that had been incubated for 60 days and had weight changes were selected for scanning electron observation microscopy. Observations revealed a large number of bacteria adhering to the microplastic surfaces (Figure 6), confirming the colonization of the colonies on PE and PP MPs. These bacteria were not present on the original surface of the material, indicating signs of biodegradation of the MPs. The filaments secreted by the bacteria can be seen in Figure 6B,C. Consistent with previous findings, the bacteria formed a biofilm on the surface of the microplastics when they were attached to them [67]. This suggests that the bacteria attached to microplastics have an adaptive response to the ecological niche of these microplastics and acquire the ability to degrade polymers during long-term colonization.

Changes in the surface are considered to be a distinctive sign of degradation [68]. Surface changes in treated membranes may be due to extracellular polysaccharides or enzymes produced by microorganisms that use microplastics as a carbon source [69]. Similar findings have been published by a few studies to show how the surface morphologies of PP and PE MPs treated with bacteria differ [35,66,70]. Also, as early as the 1970s and 1980s, microbial biofilms observed on materials using scanning electron microscopy (SEM) were seen as “evidence” of material deterioration or degradation, and now SEM observations could be used as additional information to support the biological nature of the transformation [34]. Therefore, we made subsequent AFM observations and FITR observations.

### 3.5. Atomic Force Microscopy Results before and after Microplastic Degradation

**Figure 7:**
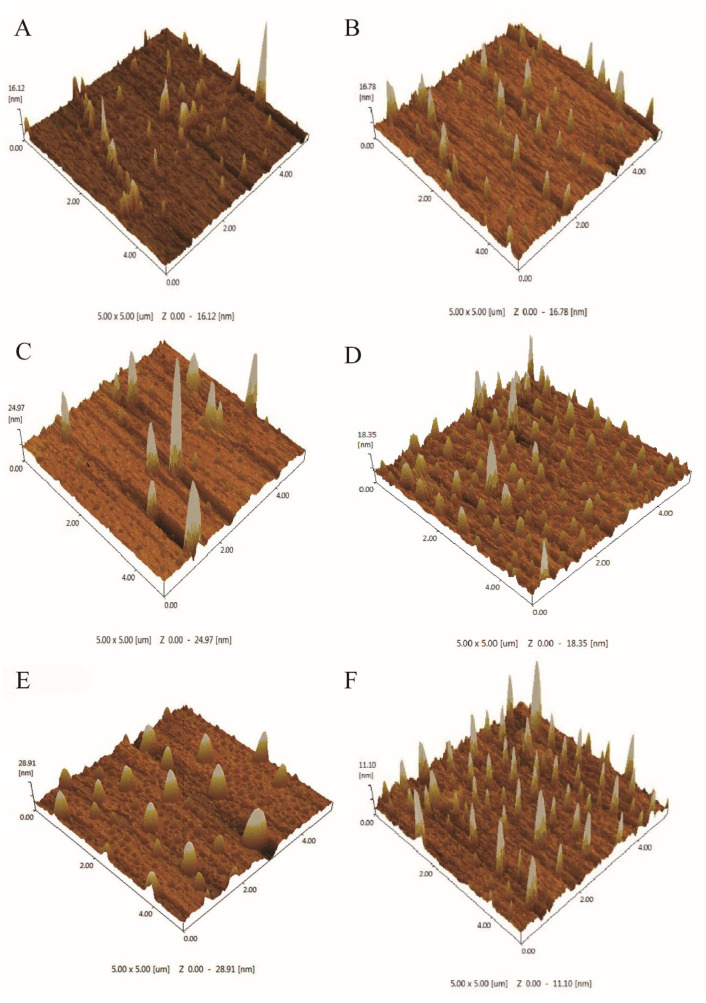
Surface microcharacterization of PE and PP microplastics before and after degradation by atomic force microscopy. PE MPs (**A**) and PP MPs (**D**) before degradation. PE MPs after degradation by GIA7 (**B**) and GIA17 (**C**). PP MPs after degradation by GIB8 (**E**) and GIB10 (**F**).

The surface characteristics of microplastics were scanned using AFM to analyze whether the surface roughness increased, and the magnitude of bulging was compared. Compared to the untreated microplastics PE (Figure 7A) and PP (Figure 7D), the surface roughness of microplastics cultured by potentially efficient plastic-degrading bacteria increased significantly. The surface of PE MPs after degradation by GIA7 was rougher and less bulging (Figure 7B), while the surface of PE MPs after degradation by GIA17 partially bulged more (Figure 7C). The surface roughness of PP MPs after degradation by GIB8 was slightly higher than that of the control group (Figure 7E), while the surface of PP MPs after degradation by GIB10 (Figure 7F) was uneven and with a large bulge. It indicated that the bacteria still had a certain degradation effect on PE and PP MPs, and the degradation effect of Bacillus cereus was better. Similar topographical alterations in polymer surfaces brought on by bacterial treatment have been observed in a number of other studies [71,72,73]. Elevated roughness values in polymer membranes treated with bacteria could be attributed to bacterial pits, cracks, and erosion [69].

### 3.6. Fourier Infrared Spectroscopy Analysis of Microplastics before and after Degradation

**Figure 8:**
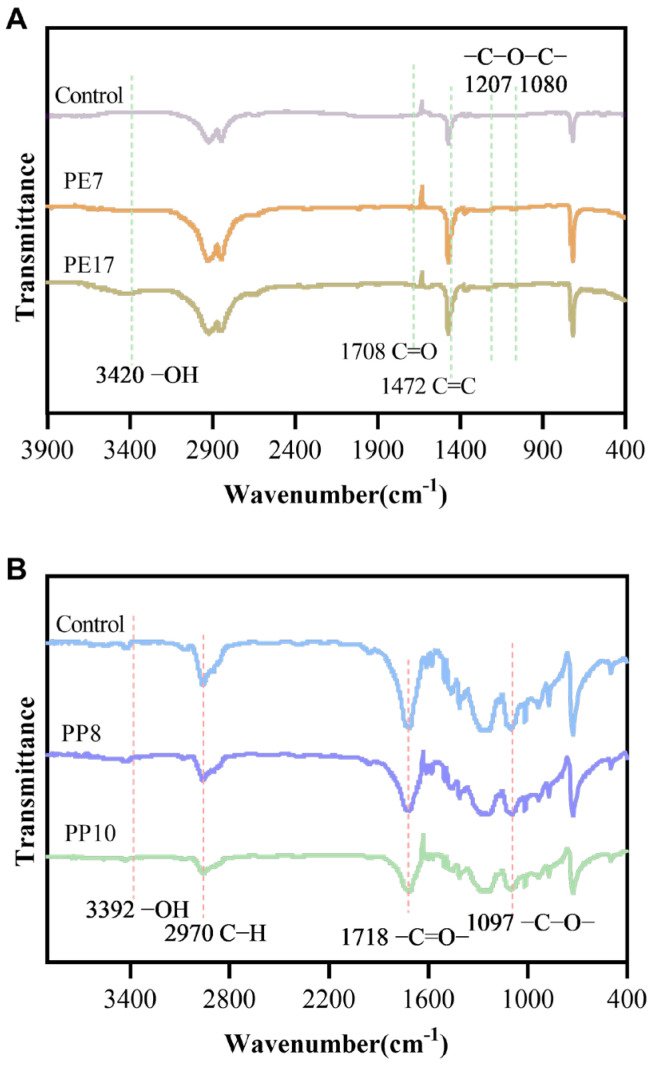
Chemical modification of PE (**A**) and PP (**B**) treated by microplastic-degrading bacteria was analyzed by Fourier infrared spectroscopy.

In the FTIR image of the control and PE MPs after degradation by GIA7 and GIA17 (Figure 8A), the peaks of C=C, a characteristic group of the PE MPs structure, appeared at 1472 cm^−1^, and the peaks of PE MPs after degradation by GIA7 and GIA17 became larger compared to those of the control. At the same time, new absorption peaks appeared in PE MPs after degradation by GIA7 and GIA17, with the formation of a polar oxygen-containing functional group hydroxyl (-OH) at 3420 cm^−1^, a carbonyl group (C=O) at 1708 cm^−1^, and an ether bond (-C-O-C-) in the region between 1207 and 1080 cm^−1^. It indicated that, after incubation of the PE MPs with the bacteria, the bacteria introduced a polar oxygen-containing functional group (C=O) into the PE MPs. The formation of these new bonds with changes in the peaks suggests that degradation of PE MPs by alcohol may have occurred after incubation of the PE MPs with the bacteria, as well as that the introduction of polar oxygenated functional groups by the bacteria into the PE MPs reduced the stability of the microplastics, favoring subsequent microbial colonization and degradation. The synthesis of enzymes by the biofilms for biodegradation on the polymer surface is revealed by the novel link creation and chemical changes in the polymer structure [74]. Numerous comparable investigations revealed that during the bacterial biodegradation of polyethylene samples, the C=O stretch occurred at various ranges, including 1749.44 cm^−1^, 1740–1690 cm^−1^, 1710–1665 cm^−1^, 1750–1735 cm^−1^, and 1790–1715 cm^−1^ [66,75]. At the same time, the addition of oxygen led to the appearance of peaks associated with C-O stretching (1000–1200 cm^−1^) and alcohol groups (R-OH stretching, 3000–3500 cm^−1^) [76]. Comparison of the infrared spectra of PE MPs after degradation by GIA7 and GIA17 showed that the intensity of polar hydroxyl (-OH) and ether (-C-O-C-) absorption peaks of PE MPs after degradation by GIA17 between 3420 cm^−1^ and 1207 cm^−1^ and 1080 cm^−1^ became larger, which indicated that GIA17 was more effective in the degradation of PE MPs. 

In the FTIR image of the control and PP MPs after degradation by GIB8 and GIB10 (Figure 8B), the characteristic peak of C-H, which is characteristic of PP MPs structure, appeared at 2970 cm^−1^, and the peak of PP MPs after degradation by GIB8 and GIB10 became smaller compared to the control. In addition, the hydroxyl (-OH-) absorption peak appeared at 3392 cm^−1^, the carbonyl (C=O) stretching vibration appeared at 1718 cm^−1^, and the -C-O- stretching vibration appeared at 1097 cm^−1^, and the intensity of the peaks of PP MPs degraded by degrading bacteria was reduced compared with that of the control. In agreement with previous findings, a hydroxyl (-OH-) absorption peak occurred at 3419 cm^−1^, a primary alcohol group (C-O) was generated at 1085 cm^−1^, and a carbonyl group (C=O) developed at 1706 cm^−1^ [77,78,79]. It has been shown that when the PP MPs surface is oxidized or passivated, the content of C and O increases, while the relative content of N decreases instead. In addition, the presence of the carbonyl group can trigger the cleavage (chain breaking) and cross-linking of the PP polymer ring, while also reducing the hydrophobic property of PP [80,81]. Park et al. [82] conducted biodegradation experiments of PE using a hybrid polyethylene degrader consisting of *Bacillus* and *Bacillus sphaericus* and obtained bond stretching results. Meng et al. [83] used *Pseudalkalibacillus* sp. MQ-1 to degrade PE, and observed that new -OH and -C=O functional groups were produced, reflecting the oxidative change of the PE chemical structure during PE degradation. Auta et al. [84] used *B. gottheilii* to degrade PP and observed peak shifts and formation of oxidation products that altered the polymer through oxidation reactions. The changes in these peaks observed in our experiments suggest that the same mechanism of biodegradation of PE and PP MPs was also described in earlier articles [85,86]: oxidation, chain cleavage, and subsequent attachment of groups containing oxygen, which modifies the structure of the polymer through hydrolysis or oxidative cleavage pathways.

## 4. Conclusions

In this study, four strains of bacteria with the potential to biodegrade PE and PP were isolated from mangroves. The strains degraded MPs at varied speeds depending on the environmental variables. The degradation rate increased with time, and it was higher in a weakly acidic environment and at a salt content of 2 g/L. The appropriate addition of Mg^2+^ and K^+^ may enhance the degradation caused by bacteria strains. From the quantitative and qualitative analysis of enzyme activities, bacterial lipases showed strong activity in strains GIA7, GIA17, GIB8, and GIB10, as well as relatively strong activity of laccase, which was shown in GIA7 and GIA17. The results of SEM, AFM, and FTIR analyses confirmed that there were significant surface changes and chemical modifications of PE and PP MPs treated with the isolate strains. This study provides potential candidate strains for biodegradation to possibly eliminate white pollution from ecosystems. Mangrove forests, as coastal intertidal zones, deposit a lot of microplastics from both land and sea, and future research could further investigate screening strains on other plastic types (e.g., PET) and optimize plastic degradation on practical applications.

## Figures and Tables

**Table 1:** Basic information on potentially efficient degrading bacteria.

Isolate Codes	Identified Bacteria	Similarity (%)	NCBI Accession No.
GIA7	*Pseudomonas* sp.	100	KX594418.1
GIA17	*Bacillus cereus*	99.33	OQ842280.1
GIB8	uncultured *Acinetobacter* sp.	100	KU942477.1
GIB10	*Bacillus cereus*	99.18	OQ788311.1

## Data Availability

The original contributions presented in the study are included in the article/Supplementary Materials, further inquiries can be directed to the corresponding author/s.

## Supplementary Materials

The following supporting information can be downloaded at: https://www.mdpi.com/article/10.3390/microorganisms12102005/s1, Figure S1: Weight loss rates of PE or PP under the degradation of 4 strains of PE and PP microplastic-degrading bacteria; Table S1: Composition of microcarbon medium utilized in the biodegradation assay; Table S2: Composition of inorganic salt medium utilized in the biodegradation assay; Table S3: Laccase, urease, lipase, H_2_S, and H_2_O_2_ production tests; Table S4: the medium indicator reaction results of PE and PP MP-degrading bacterial strains.
