# Supplementary material for: Isolation and Identification of Four Strains of Bacteria with Potential to Biodegrade Polyethylene and Polypropylene from Mangrove
Source: Microorganisms. 2024 Oct 2;12(10):2005. doi: 10.3390/microorganisms12102005 (PMC11509307; doi:10.3390/microorganisms12102005)
Supplement: Supplementary file 1 [file microorganisms-12-02005-s001.zip › microorganisms-3193912-supplementary.pdf]

Supplementary

Table S1 Composition of microcarbon medium utilized in the biodegradation assay.

S.No.	Microcarbon	Concentration (g/L)
1	peptone	0.6
2	NaCl	0.1
3	Potassium dihydrogen phosphate	0.1

Table S2 Composition of inorganic salt medium utilized in the biodegradation assay.

S.No.	Inorganic salt medium	Concentration (g/L)
1	Potassium dihydrogen phosphate	2
2	Ammonium chloride	1
3	Dipotassium hydrogen phosphate	7.5
4	Sodium chloride	0.5
5	Magnesium chloride	0.1

Table S3 Laccase, urease, lipase, H_2S, and H_2O_2 production tests

S.No.	Test	Methodology
1	H_2O_2 production test	Add 3% H_2O_2 dropwise directly to a colony
2	Laccase A production test	Add 2.5 g of guaiacol, 20 g of dextrose, 10 g of yeast paste, and 20 g of agar to the solid medium
3	Laccase B production test	0.03 g of RB Blue 0.03 g, sodium chloride 10 g, yeast paste 5 g, tryptone 10 g, agar 20 g
4	Urease production test	Add glucose 1 g, sodium chloride 5 g, peptone 1 g, potassium dihydrogen phosphate 2 g, 0.4% phenol red solution 3 mL in solid medium
5	Lipase production test	Add glycerol tributyrate 1 mL, sodium chloride 10 g, peptone 10 g in solid medium, yeast extract 5 g, agar 20 g
6	H_2S production test	Add ferric citrate 0.5 g, peptone 10 g, dipotassium hydrogen phosphate 1 g, agar 20 g

Table S4 The medium indicator reaction results of PE and PP MPs degrading bacterial strains.

**Name**	**Urease**	**Laccase A**	**Laccase B**	**Lipase**	**H_2S Producing**	**H_20_2 Enzyme**
GIA1	++	-	+++	+	-	+
GIA2	++	-	++	++	-	+
GIA3	+	-	+	+	-	+
GIA4	++	-	++	+	-	+
GIA5	++	-	+	+	-	+
GIA6	++	-	++	++	-	+
GIA7	++	-	+++	++	-	++
GIA8	++	-	+	+	-	++
GIA9	++	-	+++	++	-	+
GIA10	++	-	++	++	-	+
GIA11	+++	-	+	+	-	+
GIA12	-	-	-	-	-	+
GIA13	-	-	-	-	-	+
GIA14	-	-	-	-	-	+
GIA15	-	-	-	-	-	+
GIA16	-	-	-	-	-	+
GIA17	+	-	+++	+	-	++
GIA18	-	-	-	-	-	+
GIA19	-	-	-	-	-	+
GIA20	-	-	-	-	-	+
GIA21	-	-	-	-	-	+
GIA22	-	-	-	-	-	+
GIA23	-	-	-	-	-	+
GIA24	-	-	-	-	-	+
GIA25	-	-	-	-	-	+
GIA26	-	-	-	-	-	+
GIA27	-	-	-	-	-	+
GIA28	-	-	-	-	-	+
GIA29	-	-	-	-	-	+
GIA30	-	-	-	-	-	+

Table S4 (continued)

Name	Urease	Laccase A	Laccase B	Lipase	H_2S Producing	H_2O_2 Enzyme
GIB1	+++	-	++	+	-	+
GIB2	++	-	++	++	-	+
GIB3	+	-	+	+	-	+
GIB4	++	-	++	+	-	+
GIB5	++	-	+	+	-	++
GIB6	++	-	++	++	-	+
GIB7	++	-	+++	++	-	+
GIB8	+++	-	-	+	-	+
GIB9	++	-	+++	++	-	+
GIB10	++	-	++	++	-	++
GIB11	+	-	++	++	-	+
GIB12	+	-	++	++	-	+
GIB13	++	-	+	+	-	+
GIB14	++	-	+	+	-	+
GIB15	++	-	+	++	-	++
GIB16	+	-	+	+	-	+
GIB17	++	-	+++	++	-	++
GIB18	-	-	-	-	-	+
GIB19	-	-	-	-	-	+
GIB20	-	-	-	-	-	+
GIB21	-	-	-	-	-	+
GIB22	-	-	-	-	-	+
GIB23	-	-	-	-	-	+
GIB24	-	-	-	-	-	+
GIB25	-	-	-	-	-	+
GIB26	-	-	-	-	-	+
GIB27	-	-	-	-	-	+
GIB28	-	-	-	-	-	+
GIB29	-	-	-	-	-	+
GIB30	+	-	+++	+++	-	+

#Degradation activity: “+++”, strongly positive; “++”, positive; “+”, weakly positive; “-”, negative.

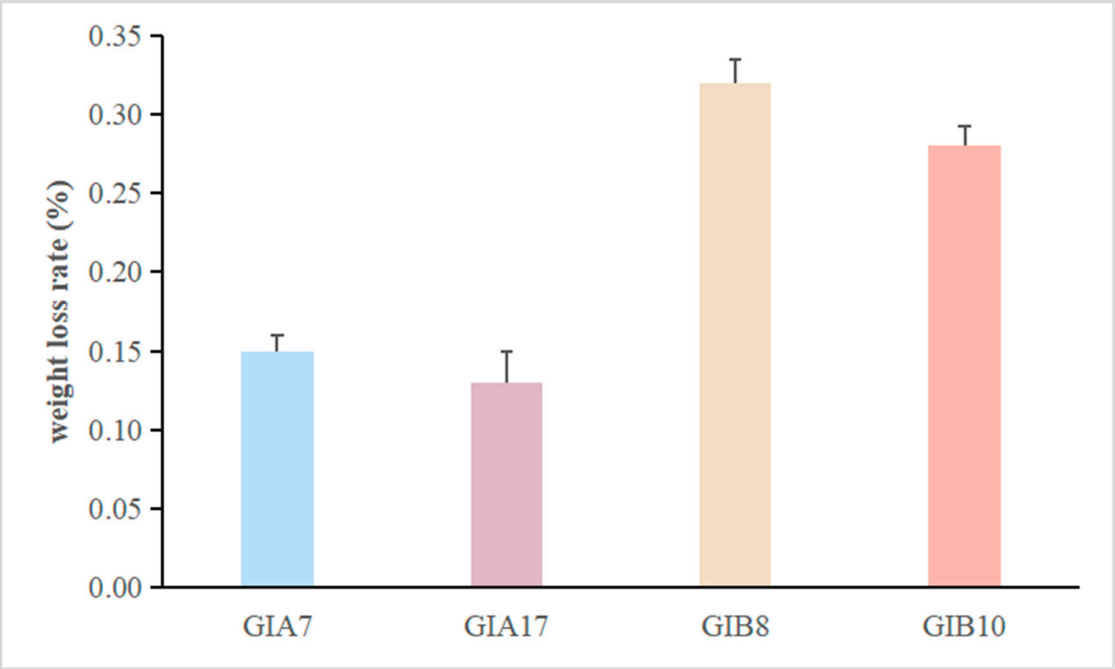

Figure S1. Weight loss rates of PE or PP under the degradation of 4 strains of PE and PP microplastic degrading bacteria.
